# Differential expression of lipoprotein genes in *Mycoplasma pneumoniae *after contact with human lung epithelial cells, and under oxidative and acidic stress

**DOI:** 10.1186/1471-2180-8-124

**Published:** 2008-07-23

**Authors:** Katri M Hallamaa, Sen-Lin Tang, Nino Ficorilli, Glenn F Browning

**Affiliations:** 1 Department of Veterinary Science, The University of Melbourne, Parkville, Victoria 3010, Australia; 2 Microbial Biodiversity Laboratory, Research Center for Biodiversity, Academia Sinica, NanKang, Taipei, 11528, Taiwan

## Abstract

**Background:**

*Mycoplasma pneumoniae *is a human pathogen that is a common cause of community-acquired pneumonia. It harbours a large number of lipoprotein genes, most of which are of unknown function. Because of their location on the cell surface, these proteins are likely to be involved in the bacterial response to environmental changes, or in the initial stages of infection. The aim of this study was to determine if genes encoding surface lipoproteins are differentially expressed after contact with a human cell line, or after exposure to oxidative or acidic stress.

**Results:**

Using qRT-PCR assays, we observed that the expression of a number of lipoprotein genes was up-regulated when *M. pneumoniae *was placed in contact with human cells. In contrast, lipoprotein expression was generally down-regulated or unchanged when exposed to either hydrogen peroxide or low pH (5.5). When exposed to low pH, the mRNA levels of four polycistronically transcribed genes in Lipoprotein Multigene Family 6 formed a gradient of decreasing quantity with increasing distance from a predicted promoter.

**Conclusion:**

The demonstrated transcriptional changes provide evidence for the functionality of these mostly unassigned genes and indicate that they are regulated in response to changes in environmental conditions. In addition we have shown that the members of Lipoprotein Gene Family 6 may be expressed polycistronically.

## Background

*Mycoplasma pneumoniae *is one of the most common causes of community acquired pneumonia [1]. It is thought to be an extracellular pathogen that colonizes the surface of epithelial cells, although there are reports suggesting that it is capable of invading host cells [2,3]. Mycoplasmas lack a cell wall, and, hence, their membrane-bound proteins are exposed to the external environment and to their host's immune response. In spite of their apparent vulnerability to immune responses, they establish chronic infections.

The *M. pneumoniae *genome has a high proportion of genes predicted to encode lipoproteins (6.68% of all genes) and 67 of these are members of six lipoprotein gene families [4,5]. A full description of these gene families, including their genomic locations, has been published previously [5]. The functionality of these genes is unknown, but because their products are cell surface proteins they could potentially play a role in adhesion and/or the initial stages of infection. The members of Lipoprotein Gene Family 2 are homologs of an *M. hyopneumoniae *lipoprotein located immediately upstream of a conserved ABC transport operon containing an exonuclease [6]. Little is known about general stress responses in *M. pneumoniae*, with only one study exploring the effects of heat shock [7], and another reporting changes in the global protein synthesis pattern in response to the presence of glycerol [8]. It has been suggested that the genome lacks many of the transcriptional regulators found in other bacteria. For example, no two-component regulatory system and only one sigma factor has been identified [4,9]. Hence, the extent of transcriptional regulation and the mechanisms involved remain largely unknown in this organism.

Understanding the changes in lipoprotein gene expression in response to different environmental conditions is an important first step in elucidating the possible functions of these genes and their transcriptional regulation in *M. pneumoniae*. Genes that are regulated after contact with host cells or under oxidative stress may play an important role in pathogenesis. In this study we used quantitative reverse transcriptase polymerase chain reaction assays (qRT-PCR) and a cell culture model employing the human lung carcinoma epithelial cell line A549 to examine changes in lipoprotein gene expression in *M. pneumoniae *in response to association with host cells. The A549 cell line has been used in previous studies of adhesion, infection or internalization by *M. pneumoniae *[3,10-12]. We also investigated changes in lipoprotein gene expression after oxidative stress or exposure to low pH, mimicking host defence mechanisms the bacteria may encounter upon infection. In total, 28 genes were chosen as representative of all lipoprotein genes and their response to changes in environmental conditions was investigated (Table 1). These 28 genes were selected to include at least one full-length ORF from each phylogenic group within the lipoprotein gene families [5].

**Table 1:** Oligonucleotides used for qPCR

Family	Gene annotation (NCBI)	Forward primer	Reverse primer
HKG^a^	MPN430	GACGTATTGGACGCCTTGTT	GTCGTTAACGGCAACGATCT
HKG	MPN665	GGCAAGAACACCCCTATT	TAGGATCACCTTCAAGCG
HKG	MPN003	GGGGAAGTGATTGGTGAT	TTGGTACCGTGTTCCTTG
			
1	MPN084	AACCCATTTCTTTGCATTGG	GTTTTGGGGATTGCTGATGT
	MPN591	ACACAACTGTGGGAATGCAA	CCCTGCCTTAGGTTTTTGGT
	MPN592	TGCTTGCTCTGCTACGCTAA	GCTCAATGGTGGTTGAGGTT
	MPN083	ACCAACGGTTTTTGAACAGC	GCATCATTGGGGTTGGATAG
	MPN588	AAAGCCTTGGTTTCGATTCA	TGCCAGGAAGACTTGTTGTG
	MPN582	TGTGCGAACCGTTGATTTTA	CTAATTTGCTTGGTGCGACA
			
2	MPN199	AGTTTCCGCTAGTTCGTTGC	GTTTTTGCGGCATCTTCAAT
	MPN408	ATTCCCATTTCCCTTTCCAC	CATTTGAGCACCGTTTTCCT
	MPN200	TTCCGGTCTCTGTTTCGACT	TCTTTTTGTGCGCCCTTACT
	MPN152	CGATTAATGGACCCGTTTTG	TCTTTGCACCGAAGTGACAG
			
3	MPN436	CCCAGTCAAGGGTTAGGTCA	TCTTCGGCAAAGAAAGGAAA
	MPN444	AACCGAAGTCAAAAACCC	GAAGTGTCATCAGCAGCC
	MPN489	GATGGTAGTTACCCCGCT	ACTAAAGCGGCAGATCCT
			
4	MPN456	AGCTGCACAAAGAAGCAC	CTTGAGTGCCGTTACCAC
			
5	MPN011	AAAGGCATTAGCGATGTTTCA	AATGTTTGTCACCTTTGTGGA
	MPN012	AGAGTGCGGAAAAAGGTGAA	AAAGGATCATTGCCTGTTGC
	MPN411	GGTATTGCGGAACTTGCTTT	TCAACTTTCCGCTCCATTTT
	MPN271	TGCGGATTTTGATTTTGACC	GATCAACCTTTCGCTCCATC
	MPN505	TTTGAAAAGGGCGAATTAGG	TGATCAACCTTTCGCTCAAA
			
6	MPN647	ATGGATCCTTCCCGTTTTTC	CCGGGATAAGTTTCTGCAAG
	MPN646	TGAACTGGGCGATAAGGAAG	AACAAATTTGAAGCAGGTGGA
	MPN645	TGGCGGAGTAAAAGAAACTGA	TCAAAAGAAGTGGCACCAAA
	MPN644	GGAGTGCAAAGCCAAAAAGT	CTTCACCACTGCCAACAATG
	MPN643	TGGCAGAAGCATTGAAGATG	TGTGTGTATCTTTCCACCAAGC
	MPN642	CCACATAAAGATGGAAGGGATG	TCCAGTATATTCACTTTCTTCTACGC
	MPN641	TCAATTGAGGGAAGGCGTTA	TGCAGTGACTCCACAAAAGC
	MPN640	TTCGGGAGGTAAAGGTAGCA	TGAATTTGGCTTTTTCACCA
	MPN639	CGCGAAAGTTACGGCTTAAA	AAGCGGCCTACTTCAGTTTG

^a ^housekeeping gene

## Methods

### Bacterial strain and culture conditions

*M. pneumoniae *strain M129 was cultured in 150 cm^2 ^tissue culture flasks (Falcon, Becton Dickinson) in Modified Hayflick Medium [15] supplemented with 20% horse serum (Sigma H-1138) at 37°C until a colour change was seen in the medium (1–2 days).

### The human cell line and culture conditions

The human lung cell line A549 was initially grown in flasks at 37°C in Minimum Essential Medium (MEM) supplemented with 5% fetal calf serum (FCS), 5 mM sodium hydrogen carbonate, 10 mM HEPES (pH 7.4) and ampicillin (50 μg/ml). The cells were subsequently cultured in 6-well tissue culture plates at 37°C in Dulbecco's Modified Eagle's Medium (DMEM) supplemented with 5% FCS, 44 mM sodium hydrogen carbonate and ampicillin (50 μg/ml) in 5% CO_2_/air, or in Leibovitz's L15 medium supplemented with 5% FCS, 10 mM HEPES (pH 7.4) and ampicillin (50 μg/ml) in air.

### Infection model

*M. pneumoniae *M129 cultured in a 150 cm^2 ^cell culture flask were scraped from the plastic into MEM supplemented with 100 U penicillin/ml. The suspension was passed through a 27-gauge needle to separate the cells.

Two millilitres of fresh MEM containing 5% FCS was added to the wells containing confluent cultures of A549 cells and 250 μl of the mycoplasma suspension was added to each well (an approximate multiplicity of infection of 100). As a control, the mycoplasma suspension was added to a well containing MEM supplemented with 5% FCS but not cells. The plates were incubated at 37°C for 15 min, 30 min, 1 h or 2 h. All time points were chosen to be less than the doubling time for *M. pneumoniae *to ensure that replication did not affect interpretation of the assay. Following incubation the medium was removed and the wells were washed twice with 2 ml of PBS at 37°C to remove non-adherent mycoplasmas. A 600 μl volume of RLT buffer (RNeasy kit, Qiagen) was added to each well and the cells were collected using a cell scraper. The cell lysate was passed through a 20-gauge needle at least 5 times and RNA was isolated using a Qiagen RNeasy kit as per the manufacturer's instructions. RNA was eluted in 50 μl of DEPC-treated water and stored at -70°C. Three independent experiments were carried out, each containing three biological replicates.

### H_2_O_2 _stress test

*M. pneumoniae *M129 was cultured in 25 cm^2 ^cell culture flasks. The cells were then incubated for 10 min in Modified Hayflick Medium at 37°C supplemented with 1 mM or 20 mM H_2_O_2_. No H_2_O_2 _was added to the negative control. The medium was discarded and RNA was isolated as described above. This experiment was repeated four times.

### Low pH stress test

*M. pneumoniae *M129 was cultured in 25 cm^2 ^cell culture flasks. The cells were then incubated for 10 min in Modified Hayflick Medium, with the pH adjusted to 5.5 or 7.4 (control) at 37°C. The medium was discarded and RNA was isolated as described above. This experiment was repeated three times.

### DNase treatment of RNA

RNA was treated with DNase prior to RT-PCR. A 15 μl aliquot of RNA was treated with 2 U of RNase-free DNase I (Novagen) in a final volume of 25 μl at 37°C for 30 minutes. The reaction was stopped by the addition of 50 mM EDTA to a final concentration of 4.5 mM, followed by incubation at 70°C for 5 minutes.

### Reverse transcription

Reverse transcription was carried out by incubating 0.5 μl random primers (3 μg/μl, Invitrogen), 1 μl dNTPs (10 nM each) and 10 μl DNase-treated RNA at 65°C for 10 minutes, and then adding 2.5 μl 10× reaction buffer (F-570B, Finnzymes), 0.5 μl RNase inhibitor (RNAguard, Amersham Biosciences), 0.5 μl AMV-RT (F-570S, Finnzymes) and DEPC-treated water to a final volume of 25 μl. The reaction was incubated for 10 minutes at 25°C, 30 min at 42°C, and then 2 min at 94°C. The cDNA was diluted 1:10 with distilled water and used as template for qPCR. As a control for detecting possible DNA contamination in samples, a duplicate reverse transcription reaction for each sample was performed without the addition of AMV-RT. The control reactions were treated identically to samples in the subsequent dilution step and in qPCR.

### Quantitative PCR

The qPCR reactions were carried out using a Stratagene MX3000P^® ^System and Platinum SYBR Green qPCR SuperMix-UDG (Invitrogen) chemistry. Expression ratios were calculated using REST 2005 [16]. Reactions consisted of 1× Platinum SYBR Green qPCR SuperMix-UDG containing the ROX reference dye at a final concentration of 50 nM, 0.75 μM forward primer, 0.75 μM reverse primer, 5 μl of template and distilled water to a final volume of 20 μl. The reactions were incubated at 50°C for 2 min, then 95°C for 5 min, then 40 cycles of 95°C for 30 s and 60°C for 30 s. Melting curve analysis was performed at the end of the amplification.

The qPCR efficiency for each primer pair was determined by running a tenfold dilution series in triplicate using M129 genomic DNA as template, at concentrations ranging from 0.5 pg to 5 ng per reaction, covering a cycle threshold (CT) range from approximately 15 to 30. The specificity of each primer pair was confirmed by melting curve analysis and subsequent agarose gel electrophoresis of qPCR products. All primer pairs produced a PCR product with a single peak in melting curve analyses and a single band after gel electrophoresis. Oligonucleotide primer sequences used in this study are provided in Table 1.

Three housekeeping genes, for glyceraldehyde-3-phosphate dehydrogenase (*gap*, also known as MPN430), elongation factor Tu (*tuf*, also known as MPN665) and DNA gyrase subunit B (*gyrB*, also known as MPN003), were validated in an adhesion assay by ensuring that the relative expression of each gene remained constant before and after contact of *M. pneumoniae *M129 cells with A549 cells. Two housekeeping genes were used for normalisation when analysing H_2_O_2_-treated samples, glyceraldehyde-3-phosphate dehydrogenase (*gap *or MPN430) and elongation factor Tu (*tuf *or MPN665). The expression of *gyrB *was not stable after exposure to H_2_O_2_. The expression of all three housekeeping genes was stable after exposure to low pH, so all three genes were used for normalisation of low pH stress samples.

### Statistical analysis

Statistical analysis of qPCR data was carried out using the randomisation test included in REST 2005 [16]. A *P *value of less than 0.05 was considered significant.

## Results

### Several lipoprotein genes were up-regulated after mycoplasma contact with human A549 cells

In total, six different genes in four distinct lipoprotein gene families were significantly up-regulated in *M. pneumoniae *after binding to A549 cells for 2 hours: MPN588 in lipoprotein Family 1; MPN199 and MPN200 in Family 2; MPN456 in Family 4; and MPN011 and MPN411 in Family 5 (Table 2). Of these genes, only MPN456 has sequence similarity to a gene of known function. BlastX analysis detected 61% identity with a homolog of a predicted component of a predicted oligopeptide ABC transport system (COG4166) in *M. genitalium *G-37 [5]. All of these genes appeared to be up-regulated after 15 min, 30 min and 1 h of incubation (see Additional file 1: Change in lipoprotein gene expression in *M. pneumoniae *after binding to A549 cells), although these results were not statistically significant.

**Table 2:** Changes in *M. pneumoniae *lipoprotein gene expression after contact with human A549 cells for 2 hours, or after incubation in 20 mM H_2_O_2 _or at pH 5.5 for 10 min

Lipoprotein family	Gene annotation (NCBI)	Change in expression (fold) after:					
		
		Contact with A549 cells#	*P *value	20 mM H_2_O_2 _for 10 min†	*P *value	pH 5.5 for 10 min§	*P *value
1	MPN084	1.1	0.714	0.5	0.439	1	0.910
	MPN591	1	0.966	0.8	0.645	1.6	0.275
	MPN592	0.9	0.880	0.9	0.906	0.9	0.825
	MPN083	1	0.934	1.7	0.504	1.3	0.605
	**MPN588**	**1.9**	0.002**	0.6	0.475	0.8	0.504
	MPN582	1.2	0.586	0.4	0.375	0.6	0.222
							
2	**MPN199**	**2.5**	0.003**	0.6	0.443	0.6	0.220
	MPN408	0.8	0.469	0.4	0.105	**0.2**	0.001**
	**MPN200**	**3.3**	0.006**	0.4	0.195	**0.4**	0.037*
	**MPN152**	1.2	0.677	0.2	0.104	**0.2**	0.020*
							
3	MPN436	0.9	0.802	0.4	0.094	0.9	0.836
	MPN444	1.3	0.549	0.7	0.240	0.8	0.555
	**MPN489**	1.4	0.286	**0.6**	0.033*	0.7	0.328
							
4	**MPN456**	**2.2**	0.010*	0.8	0.681	0.5	0.082
							
5	**MPN011**	**1.6**	0.044*	0.4	0.299	0.6	0.316
	MPN012	1.5	0.119	0.3	0.122	0.6	0.257
	**MPN411**	**1.9**	0.032*	0.5	0.345	0.7	0.442
	MPN271	1.2	0.638	0.6	0.451	0.9	0.794
	MPN505	0.8	0.602	0.3	0.095	1.5	0.302
							
6	MPN647	1.2	0.603	0.4	0.130	2.3	0.081
	MPN646	0.9	0.846	0.5	0.097	1.8	0.177
	MPN645	1.4	0.360	0.5	0.624	1.3	0.398
	MPN644	1.1	0.876	0.6	0.358	0.9	0.753
	MPN643	1.5	0.208	0.6	0.503	1.1	0.831
	MPN642	1.1	0.895	0.6	0.323	0.7	0.457
	MPN641	1.7	0.208	0.5	0.063	0.6	0.264
	MPN640	1	0.980	0.6	0.431	0.9	0.658
	**MPN639**	1.1	0.766	**0.3**	0.007**	0.8	0.514

* *P *value < 0.05.

** *P *value < 0.01.

# Mean of three independent experiments, each containing three biological replicates.

† Mean of four independent experiments.

§ Mean of three independent experiments.

### Two lipoprotein genes were down-regulated in response to hydrogen peroxide

Two lipoprotein genes, MPN489 in Family 3 and MPN639 in Family 6, were down-regulated when mycoplasma cells were incubated in growth medium supplemented with 20 mM H_2_O_2 _(Table 2). In addition, several genes in Family 2, MPN408, MPN200 and MPN152, MPN436 in Family 3, MPN012 and MPN505 in Family 5, and MPN646, MPN647 and MPN641 in Family 6 appeared to be down-regulated, although the results were not statistically significant. The lower concentration of H_2_O_2 _(1 mM) had no significant effect on lipoprotein gene expression (see Additional file 2: Change in lipoprotein gene expression in *M. pneumoniae *after exposure to 1 mM H_2_O_2 _for 10 minutes).

### Three lipoprotein genes were down-regulated after acid stress

Three genes in Family 2, MPN408, MPN200 and MPN152, were significantly down-regulated after the cells were exposed to pH 5.5 for ten minutes (Table 2). Interestingly, after low pH stress, the relative mRNA concentrations of four consecutive genes in Family 6, MPN647, MPN646, MPN645 and MPN644, formed a decreasing gradient (R^2 ^= 0.997). By employing oligonucleotides bridging adjacent ORFs in RT-PCR, these genes were shown to be polycistronically expressed (see Additional file 3: Four consecutive genes in Family 6, MPN647, MPN646, MPN645 and MPN644 are polycistronically expressed as shown by RT-PCR).

### Family 2 had the most diverse expression profiles under different environmental conditions

MPN200 in Family 2 was the only gene affected by more than one of the environmental conditions tested. This gene was up-regulated after contact with A549 cells and down-regulated after exposure to low pH.

## Discussion

In this study we report transcriptional changes in the expression of *M. pneumoniae *lipoprotein genes in response to contact with human epithelial cells *in vitro*, and provide further evidence of transcriptional regulation in mycoplasmas in response to environmental conditions [7,17-19]. We chose qRT-PCR as the method to study changes in gene expression. Although microarray and qRT-PCR results generally correlate well, both oligonucleotide and cDNA arrays have a tendency to underestimate the fold-change ratios of the underlying mRNAs compared to qRT-PCR [14]. Thus, qRT-PCR is more sensitive in detecting smaller changes in gene expression [13].

The hydrogen peroxide concentrations used in this study were chosen to have a minimal impact on cell viability, based on unpublished studies by Zimmermann and Herrmann [20]. A pH of 5.5 was used in the acidic stress test and was chosen to be below the pH of exhaled breath concentrate (pH 6.4) [21] but above the pH of 4.8 found in lysosomes. In this study mycoplasma cells were exposed to low pH or hydrogen peroxide for 10 minutes before changes in gene expression were measured. Similar hydrogen peroxide treatment times have been used previously in gene expression studies of *M. pneumoniae *[7,20] and *Haemophilus influenzae *[22]. In our cell culture infection model we allowed the mycoplasma cells to be in contact with eukaryotic cells for 15, 30, 60 and 120 minutes. This timeframe was chosen to investigate changes in gene expression during the initial stages of infection, but prior to any replication.

Global transcriptional changes in *M. pneumoniae *after heat shock have been studied using microarray technology [7]. Forty-seven genes were up-regulated after heat shock at 43°C, while 30 genes were down-regulated in the same conditions. Of the genes analysed in our study, only three genes showed differential expression in response to heat shock; MPN199 and MPN200 in Family 2 were up-regulated, whereas MPN591 in Family 1 was down-regulated. This is in agreement with our observation that Family 2 genes appear to be regulated in response to a variety of environmental conditions. Recently, in a microarray study of transcriptional regulation in *M. gallisepticum *associated with eukaryotic cells, 58 genes were found to be either up- or down-regulated [17]. The expression of *M. gallisepticum *genes homologous to *M. pneumoniae *lipoproteins in multigene Families 1, 2, 3, and 4 was not affected by contact with eukaryotic cells as detected by microarray.

All genes, apart from MPN456, that were differentially expressed in this study are hypothetical genes with no assigned function. The results from this study imply that these genes could be functional and that their gene products may play a role in adaptation to different environmental conditions. Two genes that belong to a gene family unique to *M. pneumoniae*, MPN011 and MPN411, were up-regulated when the cells were in contact with A549 cells, suggesting that the genes in this family could be involved in adhesion or in the initial stages of infection. MPN456, a homolog of a predicted component of a predicted oligopeptide ABC transport system (COG4166), was also up-regulated after adhesion to A549 cells. This could indicate regulation in response to a requirement for transport of substrates from the host cell or involvement in adhesion, as in *M. hominis*, in which a substrate-binding domain of a peptide transport system (OppA) mediates cytadherence [23,24]. However, further studies are needed to understand the role of these genes *in vivo *and whether the changes observed in gene expression of *M. pneumoniae *during association with human cells are due to adhesion, invasion or other interactions with the cell, although some recent reports of the ability of *M. pneumoniae *to invade human cells have been contradictory [2,3,25].

After acidic stress the mRNA of four genes in Family 6 formed a decreasing concentration gradient, in correlation with their distance from a predicted promoter [26]. By employing RT-PCR and oligonucleotides bridging the adjacent coding sequences, these genes were shown to be expressed polycistronically, contrary to our previous findings [5]. A positive correlation between mRNA levels and proximity to a promoter was also observed by Benders et al. [27] when they measured mRNA levels of the *ftsZ *gene cluster in *M. pneumoniae*. Groups of functionally related genes in *M. pneumoniae *and *M. genitalium *are often preceded by promoters. However no transcriptional terminators are located downstream of these genes to prevent follow-through of transcription to subsequent ORFs [27]. It has been proposed that there are only a few transcriptional terminators in the mycoplasma genomes [28,29] and, therefore, that the transcription of many genes may be initiated from upstream promoters located in front of other genes, not all of which are necessarily functionally related to those downstream.

## Conclusion

In summary, we have assessed transcriptional responses of *M. pneumoniae *after association with host cells and after exposure to hydrogen peroxide or low pH. A 20 mM concentration of H_2_O_2 _induced a trend towards general down-regulation of lipoprotein genes, whereas low pH had a more diverse effect on lipoprotein gene expression. Adhesion of mycoplasma cells to A549 cells resulted in up-regulation of selected genes, but the expression of most genes remained unaffected. These observations of transcriptional changes provide evidence for the functionality of these mostly unassigned genes and indicate that they may have a role in responses by *M. pneumoniae *to environmental changes.

## Authors' contributions

KMH carried out the molecular genetic studies, analysed the data and drafted the manuscript. NF cultured the eukaryotic cells. S-LT and GFB participated in the design and co-ordination of the study and helped to draft the manuscript. All authors read and approved the final manuscript.

## Supplementary Material

Additional file 1 Change in lipoprotein gene expression in *M. pneumoniae *after binding to A549 cells. Expression ratios after 15 min, 30 min and 1 h. Click here for file

Additional file 2 Change in lipoprotein gene expression in *M. pneumoniae *after exposure to 1 mM H_2_O_2 _for 10 minutes. Click here for file

Additional file 3 Four consecutive genes in Family 6, MPN647, MPN646, MPN645 and MPN644 are polycistronically expressed as shown by RT-PCR. Click here for file
