# Supplementary material for: Differential expression of lipoprotein genes in Mycoplasma pneumoniae after contact with human lung epithelial cells, and under oxidative and acidic stress
Source: BMC Microbiol. 2008 Jul 23;8:124. doi: 10.1186/1471-2180-8-124 (PMC2515320; doi:10.1186/1471-2180-8-124)
Supplement: Additional file 1 — Change in lipoprotein gene expression in M. pneumoniae after binding to A549 cells. Expression ratios after 15 min, 30 min and 1 h. [file 1471-2180-8-124-S1.pdf]

Additional File 1. Change in lipoprotein gene expression in *M. pneumoniae* after binding to A549 cells.

Lipoprotein gene family	Gene annotation (NCBI)	Change in lipoprotein gene expression (fold)* after binding to A549 cells for:					
		15 min	*P* value	30 min	*P* value	1 h	*P* value
1	MPN084	1.8	0.308	1.6	0.293	2.1	0.413
	MPN591	1.5	0.505	1.9	0.255	1.8	0.568
	MPN592	1.2	0.822	1.4	0.593	1.2	0.901
	MPN083	1.9	0.306	2.3	0.143	1.6	0.691
	MPN588	1.9	0.294	1.9	0.236	1.8	0.568
	MPN582	1.5	0.540	1.8	0.348	2	0.436
2	MPN199	2.1	0.129	2.6	0.166	2.5	0.221
	MPN408	1.6	0.429	1.3	0.607	1	0.979
	MPN200	1.9	0.219	2.4	0.191	3.2	0.069
	MPN152	1.5	0.487	2.1	0.445	1.7	0.603
3	MPN436	1.4	0.706	1	0.972	1.7	0.528
	MPN444	2	0.299	1.4	0.572	1.3	0.858
	MPN489	2	0.323	1.5	0.586	1.4	0.760
4	MPN456	1.5	0.5	2.2	0.181	2.4	0.228
5	MPN011	2.2	0.149	2.1	0.115	2.2	0.314
	MPN012	1.6	0.438	1.7	0.206	2	0.504
	MPN411	2.3	0.205	2	0.2	2.5	0.171
	MPN271	1.9	0.295	1.9	0.121	2.2	0.362
	MPN505	1	0.997	1.2	0.655	0.9	0.925
6	MPN647	1.4	0.655	1.3	0.521	1.6	0.784
	MPN646	1.1	0.897	1.2	0.790	1.3	0.822
	MPN645	1.8	0.445	1.8	0.215	2.3	0.355
	MPN644	1.2	0.826	0.9	0.821	1.3	0.912
	MPN643	1.6	0.525	1.8	0.320	2.2	0.382
	MPN642	1.5	0.564	1.4	0.571	1.5	0.808
	MPN641	2	0.363	2	0.332	2.4	0.057
	MPN640	1.2	0.827	1.1	0.837	1.3	0.826
	MPN639	1.5	0.614	1.3	0.720	1.3	0.892

* Mean of three independent experiments, each containing three biological replicates
