# Supplementary material for: Differential expression of lipoprotein genes in Mycoplasma pneumoniae after contact with human lung epithelial cells, and under oxidative and acidic stress
Source: BMC Microbiol. 2008 Jul 23;8:124. doi: 10.1186/1471-2180-8-124 (PMC2515320; doi:10.1186/1471-2180-8-124)
Supplement: Additional file 2 — Change in lipoprotein gene expression in M. pneumoniae after exposure to 1 mM H2O2 for 10 minutes. [file 1471-2180-8-124-S2.pdf]

Additional File 2. Change in lipoprotein gene expression in *M. pneumoniae* after exposure to 1 mM H_2O_2 for 10 minutes.

Lipoprotein gene family	Gene annotation (NCBI)	Change in lipoprotein gene expression (fold) after 10 min in 1 mM H_2O_2	
		Expression (fold)*	*P* value
1	MPN084	1	0.968
	MPN591	1.1	0.823
	MPN592	1.1	0.838
	MPN083	1.2	0.854
	MPN588	1.1	0.887
	MPN582	1	0.967
2	MPN199	1.1	0.918
	MPN408	0.5	0.436
	MPN200	0.8	0.808
	MPN152	0.8	0.631
3	MPN436	0.9	0.856
	MPN444	1.1	0.916
	MPN489	1	0.984
4	MPN456	1.4	0.710
5	MPN011	1.1	0.808
	MPN012	0.9	0.869
	MPN411	1.1	0.872
	MPN271	1.1	0.890
	MPN505	1.1	0.937
6	MPN647	1.2	0.804
	MPN646	1.1	0.839
	MPN645	1.4	0.659
	MPN644	1.2	0.774
	MPN643	1.3	0.709
	MPN642	1.2	0.838
	MPN641	1.1	0.904
	MPN640	1.3	0.691
	MPN639	1	0.967

* Mean of three independent experiments
