# Supplementary material for: Differential expression of lipoprotein genes in Mycoplasma pneumoniae after contact with human lung epithelial cells, and under oxidative and acidic stress
Source: BMC Microbiol. 2008 Jul 23;8:124. doi: 10.1186/1471-2180-8-124 (PMC2515320; doi:10.1186/1471-2180-8-124)
Supplement: Additional file 3 — Four consecutive genes in Family 6, MPN647, MPN646, MPN645 and MPN644 are polycistronically expressed as shown by RT-PCR. [file 1471-2180-8-124-S3.pdf]

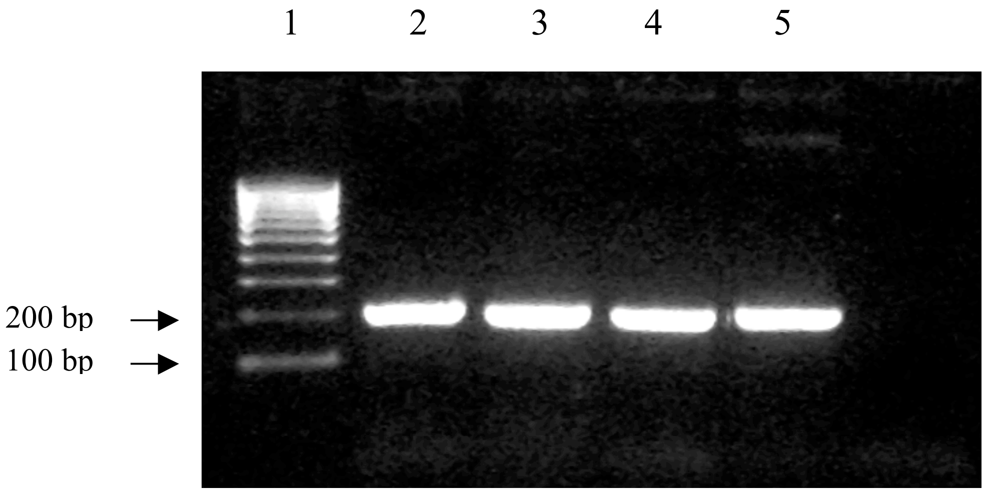

Additional File 3. Four consecutive genes in Family 6, MPN647, MPN646, MPN645 and MPN644 are polycistronically expressed as shown by RT-PCR. A positive RT-PCR result is obtained when oligonucleotides bridging adjacent ORFs are used (Lanes 2-5) to amplify *M. pneumoniae* RNA in RT-PCR. Lane 1 Bioline Hyperladder IV marker, Lane 2 MPN646-MPN647, Lane 3 MPN645-MPN646, Lane 4 MPN644-MPN645, Lane 5 MPN643-MPN644. The *M. pneumoniae* strain M129 was grown, RNA was isolated and DNAse treated, and RT-PCRs and gel electrophoresis were performed as described in Hallamaa et al 2006. The primer sequences for MPN646-MPN647 were tggctgaagttctcaattggt and aaccgctcttttaaaggcaaa, for MPN645-MPN646 ttgttgagttcaccttgactgg and gccagtgttaattacccaaacc, for MPN644-MPN645 Acattggtggcagttgatga and aatggaagccaatgaacaag, and for MMPN643-MPN644 caagcagttaaagctgttccaa and tgcaagcacaactcatgaaa.
